# Effect of Melatonin Administration on Sleep Quality in Sulfur Mustard Exposed Patients with Sleep Disorders

**Published:** 2018

**Authors:** Seyyedeh Soghra Mousavi, Majid Shohrati, Ensieh Vahedi, Meghdad Abdollahpour-Alitappeh, Yunes Panahi

**Affiliations:** a *Chemical Injuries Research Center, System Biology and Poisonings Institute, Baqiyatallah University of Medical Sciences, Tehran, Iran. *; b *Chemical Injuries Research Center, System Biology and Poisonings Institute, Baqiyatallah University of Medical sciences, Tehran, Iran.*; c *Chemical Injuries Research Center, System Biology and Poisonings Institute, Baqiyatallah University of Medical Sciences, Tehran, Iran. *; d *Basic and Molecular Epidemiology of Gastrointestinal Disorders Research Center, Research Institute for Gastroenterology and Liver Diseases, Shahid Beheshti University of Medical Sciences, Tehran, IR Iran.*

**Keywords:** Melatonin, Sleep quality, Sulfur mustard, Chronic pulmonary problems, Sleep disorders

## Abstract

Sulfur mustard (SM) is a toxic agent that targets several tissues. It is the leading cause of persistent lung disease, progressive deterioration in lung function, and mortality among injured patients. Disturbed sleep and poor quality of sleep are common in SM-exposed patients with chronic respiratory problems. Melatonin is an alternative medication that has been widely used to treat poor sleep quality caused by several specific conditions. This study aimed to evaluate the efficacy of melatonin administration in improvement of sleep quality in SM-injured patients. In this randomized, double-blind and placebo-controlled trial study a total of 30 SM-exposed male patients were recruited. Patients received 3 mg melatonin (N = 15) or placebo (N = 15), orally in a single dose, 1 h before bedtime for 56 consecutive days. Sleep quality was evaluated by Pittsburgh Sleep Quality Index (PSQI); daytime sleepiness was measured by Epworth Sleepiness Scale (ESS), and the risk of obstructive sleep apnea was determined by STOP-Bang questionnaire. Compared with placebo, melatonin administration significantly improved global PSQI score, particularly sleep latency (P = 0.03) and subjective sleep quality (P = 0.004). Mean of global PSQI score was declined significantly (P = 0.01) from 10.13 ± 3.44 to 6.66 ± 3.08 in melatonin group. No differences in ESS and STOP-Bang scores were observed between two groups. Melatonin was effective in improving global PSQI score and sleep latency, but not daytime sleepiness and obstructive sleep apnea in SM-exposed patients. Further long-term studies involving larger number of patients are needed before melatonin can be safely recommended for the management of sleep disturbances in these patients.

## Introduction

Sulfur mustard (SM) or 2, 2ꞌ-Dichlorodiethyl sulfide, is a potent alkylating agent that targets several organs especially lung, eyes, and skin tissues (1). The highest unconventional application of SM occurred in Iran-Iraq war (1980-1988) (2). During that period, it injured more than 100,000 Iranians, which one-third of them are still suffering from long-term effects (3-5). The respiratory system is the major target of SM toxicity, which occurs in a dose-dependent manner from the nasal mucosa to the terminal bronchioles (1, 6). These adverse effects are often lethal in short term, and a source of ongoing symptoms and disability in long-term (3). SM can also cause central nervous system (CNS) excitation as well as neurological effects such as headache, anxiety, fear of the future, restlessness, confusion, and lethargy, which all these factors can affect patients sleep quality (5). 

Poor sleep quality or sleepless is now considered as one of the major complaints among SM-exposed individuals. There are a lot of subjective and objective studies that describe sleep disturbances in warfare injured patients (7-9). It is probably a consequence of multiple factors including nocturnal cough, nocturnal dyspnea, use of medication, and respiratory problems (9, 10). Objective evidence of disturbed sleep, including reduced sleep efficiency, delayed sleep onset, reduced total sleep time, and frequent periods of wakefulness have been reported in these patients (6, 7, 11, 12). Hypoxemia is also believed to be a determinant of disturbed sleep in these patients (11). Respiratory problem, which occur in 50% of SM-injured patients, is one of the most important factors that causes shortness of breath and insomnia during night. An important and common systemic consequence of respiratory problems such as chronic obstructive pulmonary disease (COPD) and bronchiolitis obliterans among SM injured patients is sleep disturbances that is characterized by insomnia and poor sleep quality (10). Obstructive sleep apnea (OSA) is also reported among SM victims (13). It is a common respiratory disorder during sleep, which is characterized by apneas and associated with a reduction in blood oxygen saturation (13). Recent investigations have shown that approximately one-quarter to one-half of OSA patients are suffering from periodic limb movement disorder (PLMD) (14). It is a periodic movement disorder that occurs during sleep and characterized by repetitive leg movements, poor quality of sleep and insomnia (15). PLMD with OSA cause dopamine transmission system and sleep disturbances (16). Posttraumatic stress disorder (PTSD), which is associated with anxiety, stress, depression, and sleep disturbance, has been reported in 75% of SM-injured patients (7). Therefore, sleep problems not only associated with daytime symptoms, insomnia, and chronic fatigue among SM-exposed patients, but also these negatively affect the quality of life of these patients. As the result, administration of conventional hypnotics is not recommended in patients with respiratory failure, as these drugs may suppress ventilatory response and exacerbate sleep-related breathing disorders (17). However, benzodiazepines have been reported to increase frequency and duration of nocturnal hypoxemia in normocapnic patients (18). Therefore, a better therapeutic approach is crucial to improve the quality of sleep among these patients.

Melatonin is an endogenous hormone that is synthesized and secreted into the systemic circulation and cerebrospinal fluid by the pineal gland. It plays a crucial role in regulating the circadian rhythm and sleeping during the night and may have sleep-inducing activity in humans (17, 19). It has also immunomodulatory and antioxidant properties and its safety has been confirmed previously. Exogenous melatonin administration has been shown to improve sleep in several medical conditions (19). It does not produce a rapid increase in subjective sleepiness or major impairments in cognitive performance in contrast to common hypnotics (20). To the best of our knowledge, there is no study related to melatonin effect on sleep quality in patients with respiratory problems caused by SM exposure. Given the important role of melatonin in regulation of sleep quality, we aimed to consider the efficacy of melatonin administration in improving sleep quality in SM injured patients.

## Subjects and methods

*Study population*

The patients were individuals who had a documented encounter with SM during the Iran-Iraq war. During the study, all participants had a stable condition and complained of sleep disorder. After preliminary considerations and pulmonary function tests (PFTs), we entered patients with mild (FEV1 ≥ 80%) and moderate (50% ≤ FEV1 ≤ 80%) lung damages, which their sleep disorders were confirmed by polysomnography at sleep clinic of Baqiyatallah University of Medical Science. In this study, 14 patients were considered as mild and 16 patients were moderate in case of PFTs results. However, there was no significant difference regarding the basic demographic data and PFTs results between mild and moderate patients. We initially divided the mild patients randomly into two groups including melatonin and placebo. After that, the moderate patients were also divided randomly into two melatonin and placebo groups as well. The exclusion criteria were as follows: (1) cigarette smoking or a history of exposure to any other respiratory pollutants; (2) history of allergic rhinitis or other allergic diseases before exposure to sulfur mustard; (3) history of asthma, lung cancer and pulmonary tuberculosis, acute inflammation at upper and lower respiratory system; (4) history of drugs consumption that are associated with lung injuries; (5) history of systemic diseases or other chronic abnormalities which are associated with lung problems (such as heart disorders, kidney diseases, hepatitis, cirrhosis); (6) history of diabetes and hypertension.

*Questionnaires*

Results of the Pittsburgh Sleep Quality Index (PSQI), Epworth Sleepiness Scale (ESS), and STOP-Bang questionnaire were also available. The PSQI has seven components, each one dealing with a major aspect of sleep: 1) subjective sleep quality 2) sleep latency 3) sleep duration 4) sleep efficacy 5) sleep disturbance 6) use of sleep medication 7) and daytime dysfunction due to inadequate night sleep. Each element includes a 0-3 scale with a total score of 0-21. A total PSQI score of more than 5 indicates poor sleep quality with a sensitivity of 89.6% and specificity of 86.5% (17, 21).Component number 6 always scored zero, because patients who used hypnotic-sedative medication were not included in the study. The ESS is a subjective questionnaire outlined to specify the measure of daytime sleepiness. Patients are requested to evaluate the probability of sleeping or snoozing on a scale of growing probability from 0 (none) to 3 (high probability) for eight different conditions (watching TV, sitting and reading, sitting passive in a public place, being a passenger in a carrier for an hour without a break, lying down to rest in the afternoon whenever locations permit, sitting and speaking to someone, sitting softly after a lunch without alcohol, sitting in a carrier while stopped in traffic for a few minutes).Subject scores obtained from these eight sections are then collected to yield a multiplex score. A total score of 10 or greater is considered as excessive daytime sleepiness (17, 22).STOP-Bang is a questionnaire designed to determine the risk of obstructive sleep apnea (OSA) among patients. The sensitivity of STOP-Bang score ≥ 3 to detect moderate-to-severe OSA (AHI > 15) and severe OSA (AHI > 30) is 93% and 100% respectively. It consists of eight dichotomous (yes/no) items related to the clinical features of sleep apnea (Snoring, Tiredness, Observed apnea, high blood Pressure, BMI, age, neck circumference and male gender). The total score ranges from 0 to 8. Patients can be classified for their OSA risk based on their respective scores (23).

**Table1:** Demographic characteristics of the melatonin and placebo groups

**Parameters**	**Placebo**	**Melatonin**	***P*** **-value**
Age (Year)	51.66 ± 4.93	51.46 ± 5.86	0.92
Weight (Kg)	89.06 ± 11.37	89.06 ± 15.35	-
Height(cm)	171.66 ± 5.98	169.46 ± 5.86	0.31
BMI (Kg/m^2^)	30.24 ± 3.38	31.14 ± 5.86	0.59
Waist size (cm)	106.96 ± 8.32	105.75 ± 13.46	0.76
Neck Circumference (cm)	41.96 ± 2.90	42.10 ± 3.78	0.91
FVC (%)	61.84 ±19.17	73.09 ±17.19	0.14
FEV1 (%)	59.92 ± 18.68	74.90 ± 21.63	0.08
FEV1/FVC (%)	102.83 ± 19.05	102.54 ±18.05	0.97

BMI: Body mass index; FEV1: Forced expiratory volume in 1s; FVC: Forced volume vital capacity;

* P < 0.05 is considered as significant difference.

**Table 2:** Mean of ESS, STOP- Bang and PSQI scores before and after treatment in melatonin and placebo groups

**Parameters**		**Placebo**	**Melatonin**	***P*** **-value**
**ESS**	Before	12.06 ± 2.6	13.13 ± 3.41	0.34
	After	12.21 ± 4.94	12.33 ± 7.73	0.96
	*p*-value	0.47	0.24	
				
**STOP-Bang**	Before	5.06 ± 1.03	5.00 ± 1.19	0.87
	After	4.35 ± 1.82	3.8 ± 2.27	0.47
	*p*-value	0.09	0.22	
				
**PSQI**	Before	11.2 ± 3.29	10.13 ± 3.44	0.19
	After	9.69 ± 3.09	6.66 ± 3.08	0.04
	*p*-value	0.22	0.01	

* *P* < 0.05 is considered as significant difference; PSQI: Pittsburgh Sleep Quality Index; ESS: Epworth Sleepiness Scale.

**Table 3:** PSQI (individual components and global score) before and after treatment in melatonin and placebo groups

**Parameters**		**Placebo**	**Melatonin**	**P-value**
**Subjective Sleep quality**	Before	1.73 ± 0.45	2.06 ± 0.7	0.13
	After	1.38 ± 0.65	1.0 ± 1.04	0.27
	*p*-value	0.12	0.004	
**Sleep Latency**	Before	2.13 ± 0.74	1.66 ± 1.23	0.22
	After	2.38 ± 0.96	1.33 ± 1.37	0.03
	*p*-value	0.81	0.51	
**Sleep Duration**	Before	1.93 ± 1.09	1.93 ± 1.162	1
	After	1.46 ± 0.96	1.58 ± 1.16	0.77
	*p*-value	0.36	0.44	
**Sleep Efficiency**	Before	1.73 ± 1.22	1.06 ± 1.33	0.16
	After	1.15 ± 1.4	0.66 ± 1.15	0.35
	*p*-value	0.27	0.42	
**Sleep Disturbance**	Before	2.06 ± 0.59	1.86 ± 0.63	0.38
	After	1.92 ± 0.49	1.66 ± 0.77	0.33
	*p*-value	0.40	0.47	
**Use of Sleep Medication**	Before	0.0 ± 0.0	0.0 ± 0.0	-
	After	0.0 ± 0.0	0.0 ± 0.0	-
	*p*-value	-	-	
**Daytime Dysfunction**	Before	1.60 ± 1.12	1.53 ± 0.83	0.85
	After	1.38 ± 1.04	1.08 ± 1.24	0.51
	*p*-value	0.44	0.27	
**Global PSQI Score**	Before	11.2 ± 3.29	10.13 ± 3.44	0.19
	After	9.69 ± 3.09	6.66 ± 3.08	0.04
	*p*-value	0.22	0.01	

* *P* < 0.05 is considered as significant difference; PSQI: Pittsburgh Sleep Quality Index.

*Study design*

This is a randomized, double-blind, placebo controlled clinical trial (IRCT2015092924267N1) study that was conducted at Respiratory Department of Baqiyatallah Hospital and Chemical Injuries Research Center, Baqiyatallah University of Medical Sciences, Tehran, Iran. Here, we evaluated the effect of melatonin treatment on sleep quality of SM exposed patients. This study was conducted from April to June 2015. Accordingly, 30 SM-exposed male patients who full-filled the inclusion criteria and all three questioners were enrolled in the study and randomly divided into two groups including Placebo (n = 15) and Melatonin (n = 15). The Ethics Review Board of the Baqiyatallah University of Medical Sciences, Tehran, Iran (2014-2015) approved the study. All subjects signed an informed consent form and the procedures conformed to the guiding principles of the Declaration of Helsinki. For patients in melatonin group, 3 mg/day melatonin was prescribed one hour before bedtime for a period of 8 weeks and the other group received placebo. After 56 consecutive days treatment, the quality of sleep, daily sleepiness and the risk of obstructive sleep apnea among our patients were considered using PSQI, ESS, and STOP-Bang questionnaires. All patients were contacted via telephone twice a week to examine for any possible adverse effects and compliances. Furthermore, our patients and investigators were unaware of treatment allocation during the period of study.

*Statistical analysis*

Statistical analyses were performed using SPSS version 19.0 software (SPSS, IBM). Continuous variables were expressed as mean ± standard deviation. Data were examined for normality using Kolmogorov-Smirnov test. Unpaired Student’s t test was used for between-group comparisons of age, body mass index (BMI), baseline PSQI and ESS scores. Individual components of the STOP-Bang questionnaire were compared with sleep laboratory equivalent questions by using a nonparametric sign test. Comparisons within groups (before and after treatment) for global PSQI score, ESS score, and STOP-Bang results were made with paired Student’s test. Additionally, melatonin and placebo groups were compared with respect to global PSQI score, ESS score, and STOP-Bang results using two-way ANOVA for repeated measures. In all tests, a p-value of less than 0.05 was considered statistically significant.

## Results

*Demographic information and PFTs results*

This randomized double blind, placebo-controlled trial was conducted to assess the efficacy of melatonin administration in improving sleep quality in SM exposed patients with respiratory problems. Fifteen patients were randomized into the melatonin group and 15 into the placebo group. The mean age of the study participants was 51.56 ± 5.32 years. We didn’t find significant difference in mean of age between placebo and melatonin groups (Table 1). Demographic information of melatonin and placebo groups is presented in more details in Table 1. There was no overall difference in the baseline characteristics and PFTs results between two groups. No significant differences were also found between the two groups with respect to weight, height, BMI, waist size, and neck circumference before and after treatment (P > 0.05). The average bedtime during treatment period was the same for both groups (P > 0.05). PFTs data, including FVC, FEV1, and FEV1/FVC showed obstructive and restrictive patterns in the both placebo and melatonin groups; however, we didn’t observe any significant differences between these two groups either in before or after treatments (Table 1).

*Questionnaire results*

Table 2. depicts the mean of ESS, STOP-Bang, and PSQI scores before and after treatment in the both groups. There were no significant differences between the two groups in respect of ESS, STOP-Bang scores before and after treatment. Although mean of global PSQI score before and after study was more than 5 in both groups, we observed a significant trend (P = 0.01) for declined score from 10.13 ± 3.44 to 6.66 ± 3.08 in melatonin group. Furthermore, we didn’t find a significant decrease in the mean of global PSQI score in placebo group; however, it was significantly different between placebo and melatonin groups after treatment. Additional statistical analysis demonstrated that there was a significant difference (P = 0.04) in mean of global PSQI score after treatment between placebo (9.69 ± 3.09) and melatonin (6.66 ± 3.08) groups (Table 2).

Table 3. depicts PSQI global and sub domains score for melatonin and placebo groups before and after treatment. Subjective Sleep quality significantly improved from 2.06 ± 0.70 to 1.0 ± 1.04 (P = 0.004) in melatonin group after treatment. Although it was also declined from 1.73 ± 0.45 to 1.38 ± .65 in placebo group, this difference wasn’t significant. However, we didn’t observe any significant difference between placebo and melatonin groups before or after treatments.

We also found a significant difference (P = 0.03) in mean of Sleep Latency results after treatment between placebo (2.38 ± .96) and melatonin (1.33 ± 1.37) groups. There was no significant difference in the other PSQI components score between two groups before and after treatment (Table 3).

Additionally, improved sleep after melatonin treatment was not accompanied by any side effects; however, only two participants, one from placebo and one from melatonin group, complained of dryness of the mouth during the study period.

## Discussion

In this research, 30 SM-exposed patients who had respiratory problems along with poor sleep quality were considered in a randomized clinical trial to demonstrate the effect of melatonin treatment on sleep quality. The results of this study show that 3 mg melatonin taken 1 h before bedtime can moderately improve sleep in SM-exposed patients with mild to moderate respiratory problems. To our knowledge, this is the first study on melatonin and sleep quality in patients with respiratory problems caused by SM exposure.

There are a lot of studies that investigated the effects of melatonin on sleep quality among different patients. In a study by Shilo *et al*., (24), they carried out a research on the efficacy of melatonin treatment on sleep quality in 8 adult patients with respiratory failure caused by exacerbation of COPD or with pneumonia. Patients obtained either 3 mg of controlled-release melatonin or placebo at 10 pm. After melatonin treatment, they found a significant improvement in sleep quality and sleep duration. Accordingly, they introduced melatonin administration for sleep induction and resynchronization of biological clock for these patients. In another study by Halvani *et al*., (17), they investigated the efficacy of melatonin administration in improvement of sleep quality in 54 COPD patients. They evaluated sleep quality and daytime sleepiness by PSQI and ESS, respectively. They reported that melatonin significantly improves sleep quality in COPD patients with sleep complaints. The effects of melatonin administration on subjective sleep quality in COPD patients were also determined in a similar randomized, double-blind, and placebo-controlled study (19).Twenty-five patients gained either 3 mg melatonin (n = 12) or placebo (n = 13), orally in a single dose, 1 h before bedtime for 21 consecutive days. Sleep quality was assessed by the PSQI and daytime sleepiness was measured by the ESS. Melatonin treatment significantly improved global PSQI scores, particularly sleep latency and sleep duration; however, no differences in daytime sleepiness, lung function and functional exercise level were observed. Similarly, we didn’t find significant effects of melatonin on daytime sleepiness in our patients. Therefore, they concluded that melatonin can improve sleep in COPD. In another study by Campos *et al*., (25), effects of melatonin administration on improvement of sleep quality and pulmonary function were investigated in asthmatic patients. They deduced that melatonin treatment significantly improved the sleep quality, but it had no significant effects on peak flow and asthma symptoms. Positive effects of melatonin on sleep quality among elderly individuals were also considered. Garfinkel *et al*., (26) reported that melatonin administration improves sleep efficiency in the elderly people. Since melatonin levels fall with aging, it has been speculated that this diminution is a principal cause for poor sleep quality and sleep disturbance in the elderly (27).

Our results are in accordance with the results of these previous studies. In our study, SM-exposed patients received 3 mg melatonin (n = 15) or placebo (n = 15), orally in a single dose, 1 h before bedtime for 56 consecutive days. Melatonin treatment significantly improved global PSQI and some of its elements scores such as sleep latency; however, a trend was also observed for the positive effects of melatonin on subjective sleep quality among patients. In a study by Nunes *et al.,* (19), they showed that melatonin administration declined sleep latency in COPD patients. Interestingly, in our study sleep latency was also decreased clearly among SM-injured patients. Similarly, Zhdanova *et al*. (28, 29) in several studies reported that melatonin treatment in healthy participants reduces sleep latency and results in improved sleep efficacy. Nevertheless, in this study we couldn’t find a significant correlation between melatonin treatment and daytime sleepiness (using ESS score) in our study group, which was in agreement with other previous studies (17, 19). Moreover, we couldn’t observe a significant correlation between melatonin treatments and Stop-Bang score. None of preliminary studies considered relationship between melatonin administration and Stop-Bang score. A mean score greater than 3 suggests the increased risk of OSA among patients. In this study, the mean of Stop-Bang score among patients was 5.00 ± 1.19 before melatonin therapy that declined to 3.8 ± 2.27; however, it was not statistically significant. It may be a reason for non-significant effect of melatonin on other PSQI elements after treatment. There are also some conflicting literatures on daytime sleepiness in patients with pulmonary problems. For example, Cormick *et al*., (30) reported difficulty in getting to sleep and staying asleep and daytime sleepiness in patients with COPD. In contrast, Orr *et al*., (31) evaluated patients with COPD and chronic hypoxemia and were unable to find any evidence of daytime sleepiness, despite polysomnographic demonstration of a short sleep time and an increased number of arousals from sleep. In the current study, daytime sleepiness was observed among all SM-exposed patients who suffered from pulmonary disorder.

Therefore, based on our data and previous studies melatonin administration can be helpful for the treatment of sleep disorders among different patients suffering from sleep disturbances. However, the mechanism in which melatonin induces sleep has not been fully considered. Recent data have indicated that melatonin, when secreted in its physiological dose, is able to interrupt circadian rhythm that maintains insomnolence via a receptor-mediated pathway (19). Administration of melatonin induces rise in cGMP (Cyclic guanosine monophosphate) value, with peak levels coinciding with melatonin acrophase (19). Furthermore, peak plasma levels of melatonin and cGMP suggest a positive correlation with sleepiness, implying to an involvement of cGMP in melatonin hypnotic action (19, 32). 

In conclusion, melatonin was effective in improving PSQI score and sleep latency, but not ESS and STOP-Bang scores in SM-exposed patients. However, a trend was observed for the effects of melatonin on subjective sleep quality. Therefore, further long-term studies involving larger number of patients are needed before melatonin can be safely recommended for the management of sleep disturbances in these patients.

*Source(s) of support* : All financial funds for this study were provided by the Chemical Injury Research Center, Baqiyatallah University of Medical Sciences. The authors have no financial interests related to the material in the manuscript

## Conflict of Interest

The authors declare that there are no conflicts of interest.
